# Blood plasma and oral rinse liquid profiling for human papillomavirus in head and neck cancer – Unmasking false-positive p16 tissue cases and tracking disease dynamics

**DOI:** 10.1186/s12967-026-08248-1

**Published:** 2026-05-19

**Authors:** Matthias Mack, Romina Roesch, Jakob Rinecker, Fabian Stögbauer, Sara Krippgans, Markus Nieberler, Katharina Püchler, Cecilia Garcia-Perez, Ramona Secci, Anne Jacob, Nicole Pfarr, Katja Steiger, Carolin Mogler, Barbara Wollenberg, Jürgen Ruland, Irina A. Kerle, Markus Wirth, Christof Winter

**Affiliations:** 1 Institute of Clinical Chemistry and Pathobiochemistry, TUM University Hospital, School of Medicine and Health, Technical University of Munich, Munich, Germany; 2 TranslaTUM, Center for Translational Cancer Research, Technical University of Munich, Munich, Germany; 3 Department of Otolaryngology Head and Neck Surgery, School of Medicine and Health, Technical University of Munich, Munich, Germany; 4 Institute of Pathology, School of Medicine, Technical University of Munich, Munich, Germany; 5 Department of Oral and Maxillofacial Surgery, School of Medicine and Health, Technical University of Munich, Munich, Germany; 6 Clinic for Otorhinolaryngology, Phoniatrics and Paediatric Audiology, University Hospital RWTH Aachen, Aachen, Germany; 7 Department for Translational Medical Oncology, National Center for Tumor Diseases Dresden (NCT/UCC), a Partnership Between DKFZ, Faculty of Medicine and University Hospital Carl Gustav Carus, TUD Dresden University of Technology, and Helmholtz-Zentrum Dresden-Rossendorf (HZDR), Dresden, Germany; 8 Translational Medical Oncology, Faculty of Medicine and University Hospital Carl Gustav Carus, TUD Dresden University of Technology, Dresden, Germany; 9 German Cancer Consortium (DKTK), Partner Site Munich, a Partnership Between DKFZ and TUM University Hospital, Munich, Germany; 10 German Cancer Consortium (DKTK), Partner Site Dresden, Dresden, Germany; 11 Bavarian Cancer Research Center (BZKF), Munich, Germany

**Keywords:** Biomarker, Cell-free DNA, Circulating tumor DNA, Diagnostic specificity, Droplet digital PCR, Head and neck squamous cell carcinoma, Human papillomavirus, Liquid biopsy, Longitudinal monitoring, Oral rinse, Plasma, P16 immunohistochemistry

## Abstract

**Background:**

Human papillomavirus (HPV) is a major etiological factor in a subset of head and neck squamous cell carcinomas (HNSCC), particularly in the oropharynx, and reliable detection is critical for prognosis and treatment decisions.

**Methods:**

We applied droplet digital PCR (ddPCR) assays targeting HPV16 oncogenes (E6, E7) and the non-oncogenic gene E2 to tumor tissue, blood plasma, and oral rinse samples from 58 HNSCC patients. Results were compared with p16^INK4a^ immunohistochemistry (IHC). A cohort of 44 non-cancer controls was included. Longitudinal monitoring was performed in 33 patients with serial liquid biopsies.

**Results:**

ddPCR identified 41% of patients as HPV16-positive in tumor tissue, with higher specificity than p16^INK4a^ IHC, which yielded 10 likely false positives. Baseline liquid biopsy results were highly concordant with tissue findings, and all 44 controls tested negative. Both blood plasma and oral rinse performed reliably, though oral rinse may be particularly well suited for HPV-related HNSCC due to direct mucosal shedding. In longitudinal analyses, all initially HPV-positive patients showed HPV clearance after surgery. In two cases, loss of clearance (i.e. re-detection of HPV DNA in plasma and oral rinse) preceded clinically confirmed progression, with lead times of 87 and 122 days.

**Conclusion:**

ddPCR-based HPV testing provides high diagnostic specificity compared with p16^INK4a^ IHC and allows minimally invasive monitoring. Plasma and oral rinse represent complementary liquid biopsy sources, with oral rinse offering particular advantages for mucosal tumors. Longitudinal testing revealed early molecular recurrence, supporting its potential as a prognostic tool. While promising, the use of liquid biopsy–based HPV detection for screening at-risk but undiagnosed populations remains exploratory and requires further evaluation in larger cohorts.

**Supplementary information:**

The online version contains supplementary material available at 10.1186/s12967-026-08248-1.

## Background

According to GLOBOCAN 2022, cancers of the head and neck collectively account for approximately 770,000 new cases and 385,000 deaths worldwide each year. [1]. With a reported male-to-female incidence ratio of 3:1, head and neck cancers are the fifth most common cancer in men and the 12th most common cancer in women [1]. Head and neck cancers can be classified by the affected anatomical area: oral cavity, pharynx, larynx, oropharynx, nasal cavity and paranasal sinuses, or salivary glands. Only a small number of head and neck cancers originate from salivary glands [2]. About 90% of head and neck cancers originate from squamous cells of the mucosal epithelium in the head and neck region and hence are named head and neck squamous cell carcinoma (HNSCC). Major risk factors for developing HNSCC are tobacco consumption, alcohol consumption, and persisting infection with high-risk human papillomavirus (HPV) types (HPV16, 18, 31, and 45) that have been linked to driving oncogenesis [3, 4]. Here, a predominance of HPV type 16 can be observed in HNSCC – roughly 95% of HPV-related HNSCC are attributed to HPV16 [5]. From a clinical perspective, HPV-related HNSCC are associated with a better locoregional control of the tumor and an improved overall survival after chemotherapy or radiochemotherapy [6, 7]. As the HPV status is of prognostic significance, it is routinely assessed in HNSCCs in primary tumor tissue [8]. Use of p16^INK4a^ IHC is widely applied as a surrogate marker for transcriptionally active HPV infection due to the E7-mediated inactivation of the Rb pathway and subsequent p16 overexpression. However, its diagnostic performance is strongly site-dependent. In oropharyngeal HNSCC, p16^INK4a^ IHC shows high sensitivity and reasonably high specificity. In contrast, in non-oropharyngeal HNSCC, p16 overexpression may occur independently of HPV through alternative mechanisms such as CDKN2A alterations, Rb pathway dysregulation, or cellular senescence, resulting in substantially lower specificity. These site-specific differences underscore the need for molecular confirmation of HPV status, particularly outside the oropharynx [9–11]. In current clinical practice in Germany, HPV testing follows a stepwise diagnostic workflow. For suspected oropharyngeal HNSCC p16^INK4a^ IHC is routinely performed as the initial screening test. Additional molecular assays such as HPV DNA PCR or RNA in situ hybridization are used only in selected cases, including discordant IHC results, uncommon tumor sites, or clinical trial settings. RNA-based assays are available only in specialized centres and are not routinely performed. Importantly, no internationally harmonized guideline exists for HPV testing in HNSCC, leading to variation in diagnostic strategies across institutions and countries. This highlights the need for standardized molecular approaches, such as ddPCR, that offer high specificity and can complement surrogate markers like p16. Here, we aimed at developing a non-invasive liquid biopsy assay to detect HPV16-specific circulating genomic material in liquid biopsy samples. Liquid biopsies are a minimally invasive way to genetically classify the mutational and genetic profile of a cancer and study tumor kinetics and detect recurrence early [12, 13]. Despite constant advances in the field of liquid biopsies, only few applications have entered clinical routine yet [14, 15]. As part of our KOHACIN study, we have recruited 58 HNSCC patients, for which the HPV status has been assessed by p16^INK4a^ IHC. We aimed at detecting the HPV16 associated oncogenes E6 and E7, and the non-oncogenic gene E2 through digital droplet polymerase chain reaction (ddPCR) in cell-free DNA (cfDNA) isolated from oral rinse and blood plasma. The same assays were also applied to genomic DNA (gDNA) isolated from primary tumor tissue obtained during surgery or biopsy. In addition to screening for HPV associated genes and oncogenes, we also tested for RNA transcripts related to the HPV16 E1^E4, E2, E6, and E7 and proved the actual expression of the mentioned HPV related genes and oncogenes. Here, we used RNA from primary tumor tissue and the total RNA isolated from blood and oral rinse samples that we transcribed into complementary DNA (cDNA). In this study, we aimed to develop and evaluate a ddPCR-based approach for detecting HPV16 DNA and RNA in tumor tissue, blood plasma, and oral rinse samples. Furthermore, we aimed to assess the potential of these liquid biopsy approaches for HPV status determination and longitudinal monitoring in HNSCC patients. HNSCC patients with respect to overall survival (OS) and progression-free survival (PFS).In addition, we applied our HPV16 ddPCR assays to sequential blood and oral rinse HNSCC patient samples and demonstrated that the results obtained from these longitudinal liquid biopsies can be used to track treatment response after resection of the primary tumor and may be prognostic for an upcoming tumor progression. In our study we want to present our ddPCR based approach as (1) a diagnostic tool for HPV status determination in HNSCC, and (2) a prognostic tool for longitudinal monitoring of HPV positive HNSCC patients using liquid biopsies.

## Methods

### Study design and study cohorts

The prospective, monocentric KOHACIN study (NCT05122507) was conducted at TUM University Hospital, Technical University of Munich, between June 2017 and May 2022. Eligible participants were ≥ 18 years with confirmed HNSCC, staged by AJCC 8th edition; patients with active second malignancies were excluded. All provided written informed consent. A non-cancer control cohort included three subgroups: (i) age- and sex-matched healthy volunteers, (ii) patients with inflammatory conditions of the oral cavity or pharynx, and (iii) patients with traumatic head and neck injuries. Controls were recruited via outpatient clinics and surgical departments. Study reporting followed STROBE guidelines, with the checklist available in the supplement (Supplementary Table S1) [16].

### Patients’ sample collection and preparation

Blood and oral rinse samples were collected and processed according to standardized protocols detailed in supplemental methods (Supplementary Material S1). Briefly, one EDTA-blood tube, one blood tube for total RNA extraction, and 10 mL of oral rinse were collected and further analyzed. Two patient cohorts were defined based on sample availability: (i) the *baseline cohort*, consisting of patients with at least one liquid biopsy sample (blood plasma or oral rinse) collected at the time of surgery or up to 28 days prior, and (ii) the *baseline & follow-up cohort*, comprising patients from the baseline cohort with at least two additional postoperative liquid biopsy samples obtained.

CfDNA was isolated from 2 mL blood plasma using the *QIAamp Circulating Nucleic Acid Kit* (Qiagen, Hilden, Germany), and from 5 mL oral rinse samples using the *Quick-DNA Miniprep Plus Kit* (Zymo Research, Irvine, CA, USA). Blood total RNA was isolated from whole blood collected in *PAXgene Blood RNA tubes* (BD Biosciences, Franklin Lakes, NJ, USA) using the *PAXgene Blood RNA Kit* (Qiagen). Total RNA was isolated from 5 mL oral rinse using the *miRNeasy Mini Kit* (Qiagen). For comparison, gDNA and RNA were isolated from primary tumor tissue obtained via surgical resection or biopsy and preserved as formalin-fixed paraffin-embedded (FFPE) blocks. DNA and RNA isolation from FFPE samples was performed using the *Maxwell FFPE Plus DNA and RNA Kits* (Promega, Madison, WI, USA). Isolated RNA samples were subjected to DNase digestion using *PerfeCTa DNase I* (QuantaBio, Beverly, MA, USA) and reverse transcribed into complementary DNA (cDNA) using the *qScript™ XLT One-Step RT-qPCR ToughMix* (VWR). All isolated nucleic acid samples were subjected to quality control and fragment size assessment using the 4200 TapeStation (Agilent Technologies, Santa Clara, CA, USA) system. CfDNA was analyzed using High Sensitivity D1000 ScreenTapes and Reagents, total RNA using High Sensitivity RNA ScreenTapes, and gDNA using Genomic DNA ScreenTapes and Reagents.

### Digital droplet PCR

Our ddPCR assays were developed to detect HPV16 DNA (targeting the *E2*, *E6*, and *E7* genes) and HPV16-associated RNA transcripts (*E1^E4*, *E2*, *E6*, *E7*). Custom primers and fluorescently labeled probes were designed for each target using oligonucleotides (Integrated DNA Technologies, Coralville, IA, USA). Fluorophore-labeled probes were used for all assays. A reference ddPCR assay with a HEX-labeled probe targeting the *EIF2C1* (Protein argonaute-1) gene was included in all runs.

Primer and probe sequences, along with synthetic gene fragments (gBlocks) used for assay validation, are detailed in the supplementary material (Supplementary Table S1). The analytical sensitivity and specificity of the HPV16 ddPCR assays were evaluated using gBlocks corresponding to the respective HPV16 targets and genomic DNA from HPV16-positive (UP-SCC-154) and HPV16-negative (UD-SCC-5) HNSCC-derived cell lines. Cell lines were cultured in DMEM/F12 medium supplemented with 10% fetal bovine serum (FBS) and 1% penicillin-streptomycin under standard conditions (37 °C, 5% CO₂, humidified incubator). DNA was isolated from cell pellets using the *QIAamp DNA Mini Kit* (Qiagen), following the manufacturer’s instructions. All primer and probe sequences used in this study were checked with BLASTN against the core_nt database. Results were filtered to exclude HPV16 (taxid:333760) sequences. In all cases, no significant similarity was found. All ddPCR reactions were performed in duplicate using the *QX200 AutoDG Droplet Digital PCR System* (Bio-Rad Laboratories, Inc.). Detailed workflow parameters and cycling conditions are available in the supplement (Supplementary Table S1 and Supplementary Material S1). Fluorescent amplitude plots and droplet clustering were analyzed using a custom in-house R-based analysis pipeline. All ddPCR data are reported in accordance with the 2020 guidelines of the Minimum Information for Publication of Quantitative Digital PCR Experiments (dMIQE) (Supplementary Table S2). The ddPCR results for each patient sample analyzed and clinical data are provided as a supplement (Supplementary Table S3). Raw data, including amplitude and cluster data from the ddPCR runs are provided on the ZENODO repository (10.5281/zenodo.15731097).

### p16^INK4a^ immunohistochemistry

Routine p16^INK4a^ immunohistochemistry (IHC) was performed on 2 µm sections from FFPE primary tumor tissue using a p16 E6H4 antibody (Ventana/Roche, Tucson, AZ, USA; Roche catalogue no. 06695256001, RRID:AB_3675558) at the Institute of Pathology, Technical University of Munich. Antibody stainings were done on an automated immunostainer with an ultraVIEW diaminobenzidine (DAB) detection kit (Ventana/Roche). Germline gDNA was isolated using DNAzol regent (Thermofisher Scientific, Waltham, MA, USA) from the buffy coat of preoperative blood samples as a control. Blood and oral rinse samples from non-tumor control subjects were collected and processed under identical conditions to those of the patient cohorts. No tissue was collected from the non-tumor control subjects.

### Outcome definition

The primary endpoint of the study was progression-free survival (PFS), defined as the duration from the initiation of treatment to the earliest occurrence of any of the following events: cancer recurrence or progression (local, regional, or distant), death from any cause, or the date of the last documented follow-up. The secondary endpoint was overall survival (OS), defined as the time from treatment initiation to death from any cause. For patients who were still alive at the end of the observation period, OS was censored at the date of the last follow-up.

### Statistical analysis

Concordance between HPV16-related gene detection in liquid biopsy samples and primary tumor tissue was evaluated in a subset of patients with matched baseline plasma, oral rinse, and formalin-fixed paraffin-embedded (FFPE) tumor tissue samples. Associations between demographic and clinical variables and the detection of ctDNA in liquid biopsies were assessed using Fisher’s exact test. Positivity thresholds were based on negative controls. Samples were classified as positive when a minimum of four positive droplets were detected per ddPCR reaction. Survival analyses were performed using R with standard Kaplan-Meier and log-rank methods. Statistical comparisons between groups were conducted using the log-rank test. All analyses were conducted in RStudio (RStudio Team, 2020) with customized R scripts using the ggplot2 package (Wickham, 2016) for data visualization and survminer (Kassambara et al., 2024) for survival analysis.

## Results

### Patient characteristics

**Fig. 1:**
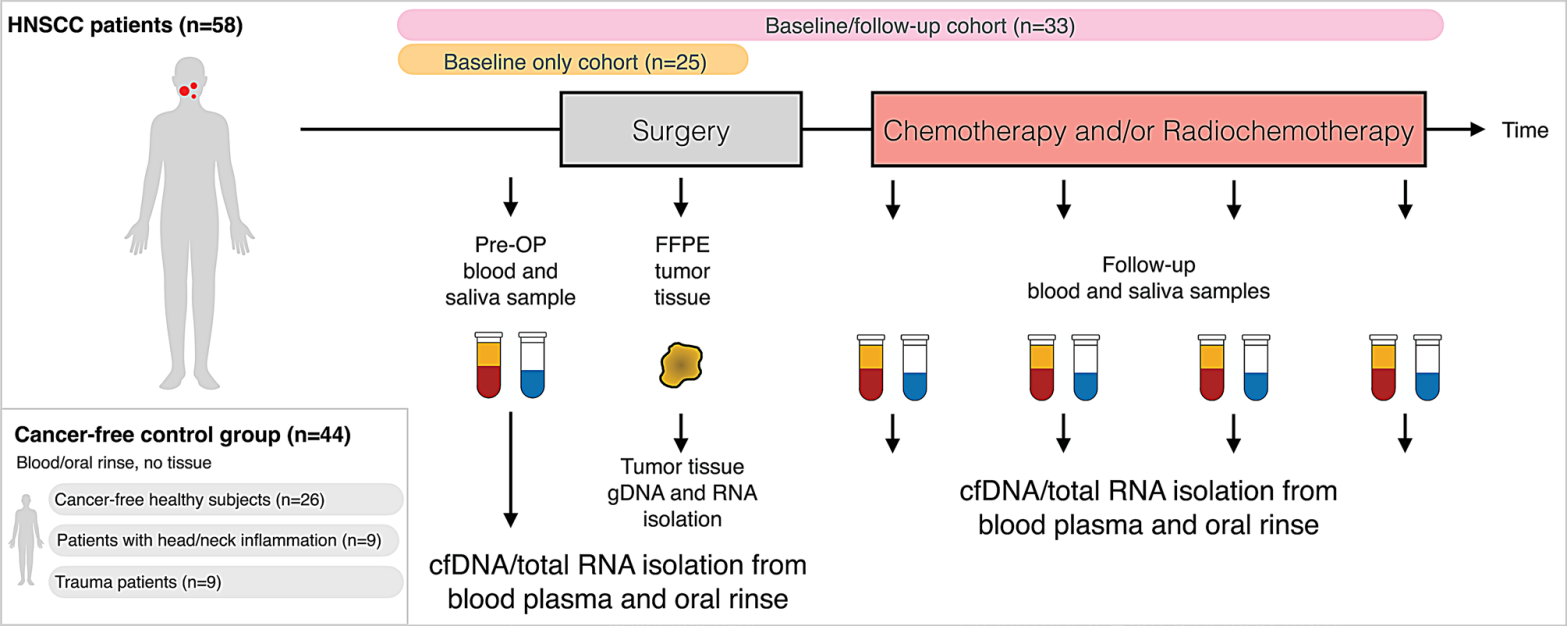
Study overview and sample collection strategy. Flow chart illustrating the definition of head and neck squamous cell carcinoma (HNSCC) patient cohorts and overall study design. The baseline only cohort (*n* = 25) included patients with at least one pre-surgical liquid biopsy (blood and/or oral rinse). The baseline/follow-up cohort (*n* = 33) included patients with baseline and at least two postoperative liquid biopsies. The control cohort (*n* = 44) consisted of healthy volunteers, patients with benign inflammatory conditions, and trauma patients

A total of 58 patients diagnosed with HNSCC were enrolled and stratified into two cohorts: the baseline cohort and the baseline & follow-up cohort. (Fig. 1). Detailed cohort information and clinical data are provided as a supplement (Supplementary Table S4). In the baseline only cohort (*n* = 25), at least one liquid biopsy sample, either blood plasma or oral rinse, was collected within 28 days prior to biopsy or surgical resection of the primary tumor. The baseline/follow-up cohort (*n* = 33) included patients where a baseline sample was available with additional liquid biopsy samples collected postoperatively, enabling longitudinal monitoring. Clinical and demographic characteristics at the time of surgery or biopsy are summarized in Table 1. The median age across cohorts ranged from 59 to 69 years, with a male predominance (69–84%) consistent with the typical gender distribution observed in HNSCC. At baseline, tumors in both cancer cohorts were primarily diagnosed at early to locally advanced stages (UICC stages I–IV), with curative intent pursued for early-stage disease. Approximately 50% of tumors were classified as HPV-positive based on routine p16^INK4a^ IHC. The oropharynx and oral cavity were the most common primary tumor sites, while fewer cases involved the hypopharynx or larynx. One case in the baseline only cohort was categorized as carcinoma of unknown primary (CUP). Patients in the baseline only cohort were followed for a median of 806 days, during which 32% experienced disease progression (median time to progression: 421 days) and 24% died (median time to death: 806 days). In the baseline/follow-up cohort, the median follow-up was 1338 days, with 42% experiencing progression (median: 360 days) and 21% dying (median: 583 days). The control cohort comprised 44 non-cancer subjects, including 26 healthy individuals recruited through outpatient general practice, 9 patients with benign inflammatory conditions of the head and neck (e.g., abscess, laryngitis, sinusitis), and 9 patients with traumatic injuries (e.g., fractures of the orbit, maxillary sinus, nasal bone, or mandible). All control participants were treated at the Departments of Otolaryngology or Oral and Maxillofacial Surgery.

**Table 1:** Patient and control cohort characteristics

	Baseline only cohort (n = 25)	Baseline/follow-up cohort (n = 33)	Control cohort (n = 44)
**Sex**			
Male	21 (84%)	24 (73%)	30 (69%)
Female	4 (16%)	9 (27%)	14 (31%)
**Age (years)**			
Median [IQR]	69 [30,78]	59 [50,74]	61 [50,68]
**p16**^**INK4a**^**Status (IHC)**			
positive	11 (44%)	22 (67%)	
negative	14 (56%)	11 (33%)	
**Localisation**			
Oropharynx	11 (44%)	22 (67%)	
Hypopharynx	1 (4%)	2 (6%)	
Larynx	1 (4%)	0 (0%)	
Lip and oral cavity	11 (44%)	9 (27%)	
CUP	1 (4%)	0 (0%)	
**Stage**			
I	6 (24%)	5 (15%)	
II	2 (8%)	15 (46%)	
III	3 (12%)	5 (15%)	
IV	13 (52%)	7 (21%)	
Unknown	1 (4%)	1 (3%)	
**Therapy**			
RTx	7 (28%)	12 (36%)	
RCTx	2 (8%)	11 (33%)	
RTx/CTx	6 (24%)	7 (21%)	
Unknown	10 (40%)	3 (9%)	
**Case outcome**			
Progression events	8 (32%)	14 (42%)	
Days until progression from first diagnosis (Median [IQR])	421 [261, 528]	360 [236, 724]	
Death events	6 (24%)	7 (21%)	
Overall survival from first diagnosis (Median [IQR])	545 [510, 718]	583 [311, 1411]	
Follow-up time (Median [IQR])	806 [585, 1469]	1338 [758, 1643]	

Demographic, clinical, and pathological features of patients with head and neck squamous cell carcinoma (HNSCC) in the baseline only cohort (*n* = 25) and the baseline/follow-up cohort (*n* = 33), alongside control subjects (*n* = 44). Characteristics include age, sex, tumor localization, p16^INK4a^ immunohistochemistry (IHC) status, tumor stage (UICC), therapy type, and clinical outcomes. IQR: interquartile range; CUP: carcinoma of unknown primary

### Sample material characteristics and HPV16 screening results based on ddPCR and p16^INK4a^ IHC

#### ddPCR based HPV16 detection rates in gDNA from primary tumor tissue

**Fig. 2:**
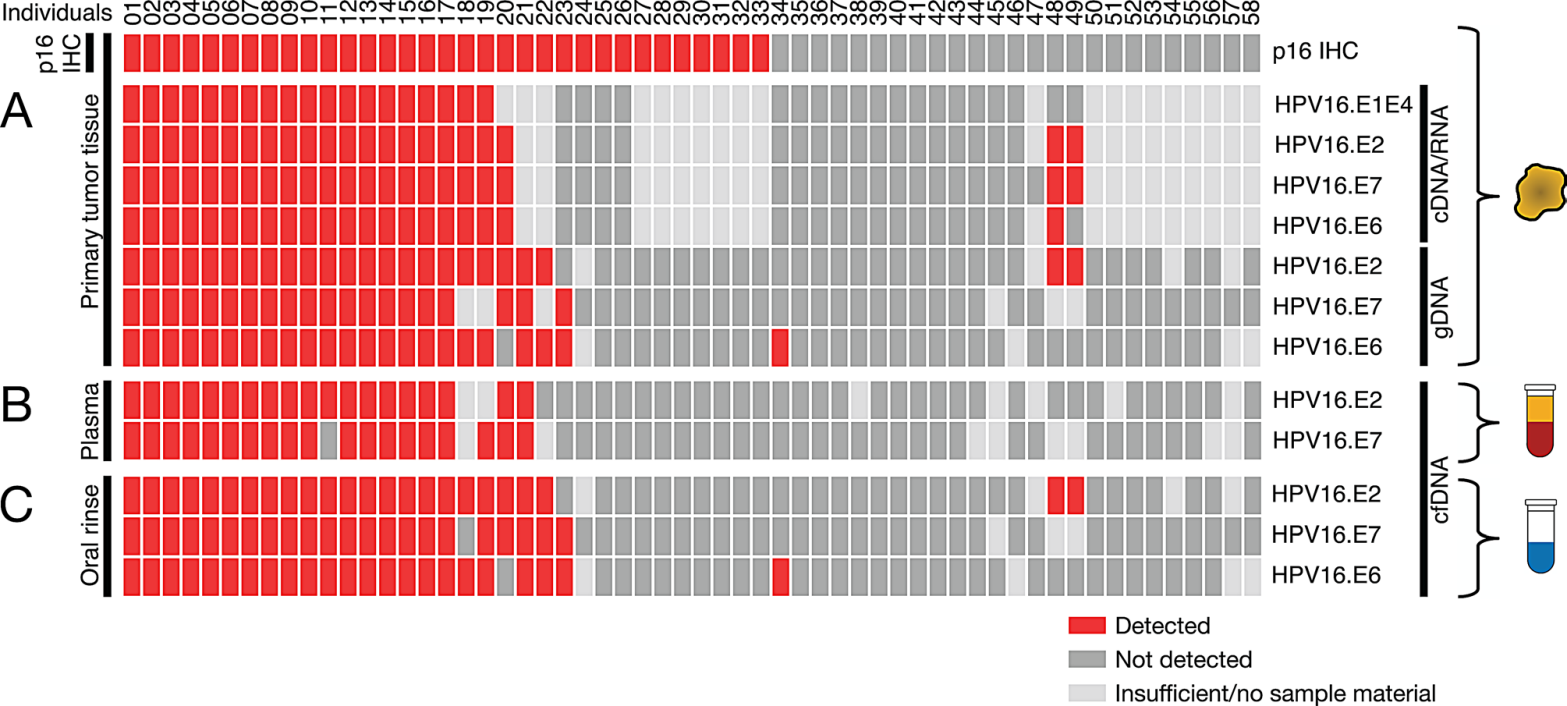
Comparison of HPV16 detection methods in tumor tissue and liquid biopsies. Heatmap comparing p16^INK4a^ immunohistochemistry (IHC) results in tumor tissue with ddPCR results for HPV16 DNA (E6, E7, E2) and RNA transcripts (E1^E4, E2, E6, E7) in tumor tissue, plasma, and oral rinse samples. Results illustrate strong concordance between ddPCR from tumor tissue and liquid biopsies, while p16^INK4a^ IHC indicates higher HPV-positivity, suggesting false-positive classifications

Primary tumor tissue obtained at baseline, either during surgery or diagnostic biopsy, was available for all 58 HNSCC patients enrolled in the study. The gDNA isolated from these samples was analyzed by ddPCR for the presence of HPV16-associated genes, including the oncogenes E6 and E7, as well as the non-oncogenic E2 gene, as illustrated in Fig. 2A. Among the samples tested, 23/54 (43%) were positive for HPV16 E6, while four samples were excluded due to insufficient DNA. For the E7 gene, 20/52 samples (38%) tested positive, with six samples omitted from testing due to low DNA quantity. The E2 gene was detected in 24/54 samples tested (44%), again with four samples not evaluated due to limited DNA availability. Using a definition of HPV16 positivity based on the detection of at least one oncogenic gene (E6 or E7), 24/58 patients (41%) were classified as HPV16-positive by ddPCR. One patient in this group was positive for E6 but negative for E7, which may reflect stochastic detection effects at low template concentrations near the limit of detection, differences in assay sensitivity between targets, or partial DNA fragmentation affecting target-specific amplification. Notably, the non-oncogenic E2 gene was also detected in the tumor gDNA of 22/24 HPV16-positive patients, supporting the robustness of the ddPCR-based classification and indicating the presence of either episomal or integrated viral DNA in the majority of cases.

#### ddPCR based HPV16 RNA detection in cDNA transcribed from primary tumor tissue RNA to proof the actual expression of HPV associated genes

To confirm the transcriptional activity of HPV16 within tumor cells, ddPCR was performed on cDNA synthesized from RNA isolated from primary tumor tissues. This approach aimed to provide molecular evidence of active viral gene expression in the tumor microenvironment. A subset of 38 patients from the total baseline cohort of 58, for whom tumor RNA was available, was analyzed for the presence of HPV16 transcripts corresponding to E1^E4, E2, E6, and E7 using the ddPCR assay. HPV16 RNA was detected in 19/38 cases (50%), indicating a substantial proportion of tumors with ongoing viral transcription. High concordance was observed across the four RNA targets, with 34/36 evaluable cases (94%) showing concordant detection across E1^E4, E2, E6, and E7 assays, further supporting the specificity of the ddPCR assay for detecting transcriptionally active HPV16 infections. Due to sample limitations, the E1^E4, E2, and E7 assays were conducted on 36 and 37 cDNA samples, respectively, rather than the full subset.

### ddPCR-Based HPV16 DNA testing identifies fewer positive cases than p16^INK4a^ IHC, with RNA evidence suggesting false positives in IHC.

Comparison of ddPCR detection of HPV16 oncogenes (E6, E7) in tumor gDNA with p16^INK4a^ IHC revealed discordance in 11/58 patients (19%). Ten cases were IHC-positive but ddPCR-negative, and one case showed the reverse. To clarify, ddPCR was also performed on cDNA that was reverse transcribed from RNA from the same tumors to assess transcriptional activity of HPV16 (E1^E4, E2, E6, E7). Results were strongly concordant with DNA findings: 37/38 matched evaluable cases (97%) showed agreement between HPV16 detection in tumor gDNA (E6 and/or E7) and RNA-based assays. In discordant cases, no HPV RNA transcripts were detected. This supports the interpretation that most IHC-positive/ddPCR-negative tumors represent false positives. Overall, ddPCR provided higher specificity than p16^INK4a^ IHC. Using ddPCR-based HPV16 detection as molecular reference standard, all 34/34 HPV-negative tumor samples were correctly classified as negative (100% specificity), whereas p16^INK4a^ IHC yielded 10 false-positive cases, corresponding to a specificity of 24/34 (71%).

#### ddPCR based HPV16 DNA detection rates in cfDNA from blood plasma, and oral rinse samples validate both sample materials as promising for HPV screening in HNSCC patients.

At baseline, liquid biopsy samples were collected from all 58 patients, including 55 blood plasma samples (Fig. 2B) and 58 oral rinse samples (Fig. 2C), obtained within 28 days prior to surgery. CfDNA was analyzed by ddPCR for HPV16-specific targets using the same assays as for tumor tissue. In plasma, HPV16 E2 was detected in 19/51 samples (37%) and E7 in 20/50 (40%). The plasma ddPCR panel was restricted to E2 and E7 because of the limited cfDNA yield obtainable from 2 mL plasma. In oral rinse, E2 was found in 23/54 (43%), E6 in 24/54 (44%), and E7 in 23/55 (42%). Applying the predefined criterion (positivity = E6 or E7 detection), 19 patients were HPV16-positive in plasma. All 19 were concordantly positive in tumor tissue and/or oral rinse, while all plasma-negative patients were negative across sample types, indicating strong cross-sample concordance. Cross-sample concordance was defined as agreement in HPV16 detection status (positive/negative) between tumor tissue, plasma, and oral rinse samples at baseline. Using this definition, all plasma-positive cases (19/19) were concordantly positive in tumor tissue and/or oral rinse, and all plasma-negative cases were concordantly negative across sample types, corresponding to complete concordance within evaluable matched samples. All 44 control subjects tested negative for HPV16 E2 and E7, confirming assay specificity. Germline gDNA from leukocyte-rich buffy coats also tested negative, excluding contamination or latent viral DNA. Together, these results validate both blood plasma and oral rinse as reliable, non-invasive sample sources for HPV16 detection in HNSCC, with excellent agreement to tumor tissue ddPCR results. To provide a patient-level overview of allele frequencies across tissue, plasma, and oral rinse, we compiled all HPV16 VAFs (E6, E7, E2) into a summary dataset (Supplementary Table S5), which also highlights OPSCC cases and enables direct inspection of inter-analyte concordance.

#### HPV16 RNA/cDNA is not detectable in total RNA from blood and oral rinse samples using ddPCR

In addition to DNA-based detection, we evaluated the feasibility of HPV RNA detection from total RNA isolated from plasma and oral rinse samples transcribed into cDNA. However, as shown in the Supplementary Figure S1, none of the RNA targets (E6, E7, and E2) could be reliably detected by ddPCR in either blood-derived or oral rinse-derived RNA/cDNA. This suggests that HPV RNA is either absent or below the detection limit of ddPCR in these sample types.

#### OPSCC-restricted diagnostic performance and concordance of ddPCR versus p16 IHC

Given the site-specific biology of HPV-driven disease, we performed an additional analysis restricted to oropharyngeal squamous cell carcinoma (OPSCC; *n* = 33). The OPSCC-specific heatmap is shown in Supplementary Figure S2, and HPV16 variant allele frequencies for all ddPCR targets are summarized in Supplementary Table S5. Within the OPSCC subset, 14 of 33 tumors were p16^INK4a^ IHC-positive. ddPCR confirmed HPV16 DNA in 9 of these 14 cases, whereas 5 p16-positive tumors showed no detectable HPV16 DNA or RNA across all assays, indicating transcriptionally inactive tumors despite p16 overexpression. One OPSCC classified as p16-negative was ddPCR-positive in tissue, plasma, and oral rinse. In liquid biopsy analyses, HPV16 DNA was detected by ddPCR in 7 of 14 p16-positive OPSCCs in plasma (with one case lacking plasma material) and in 9 of 14 cases in oral rinse. The same ddPCR-positive/p16-negative tumor was concordantly positive in both liquid biopsy analytes. These findings demonstrate improved molecular specificity of ddPCR compared with p16^INK4a^ IHC within the oropharynx and highlight oral rinse as a particularly sensitive analyte for HPV16 detection in OPSCC.

### ddPCR-based HPV16 status improves survival stratification

**Fig. 3:**
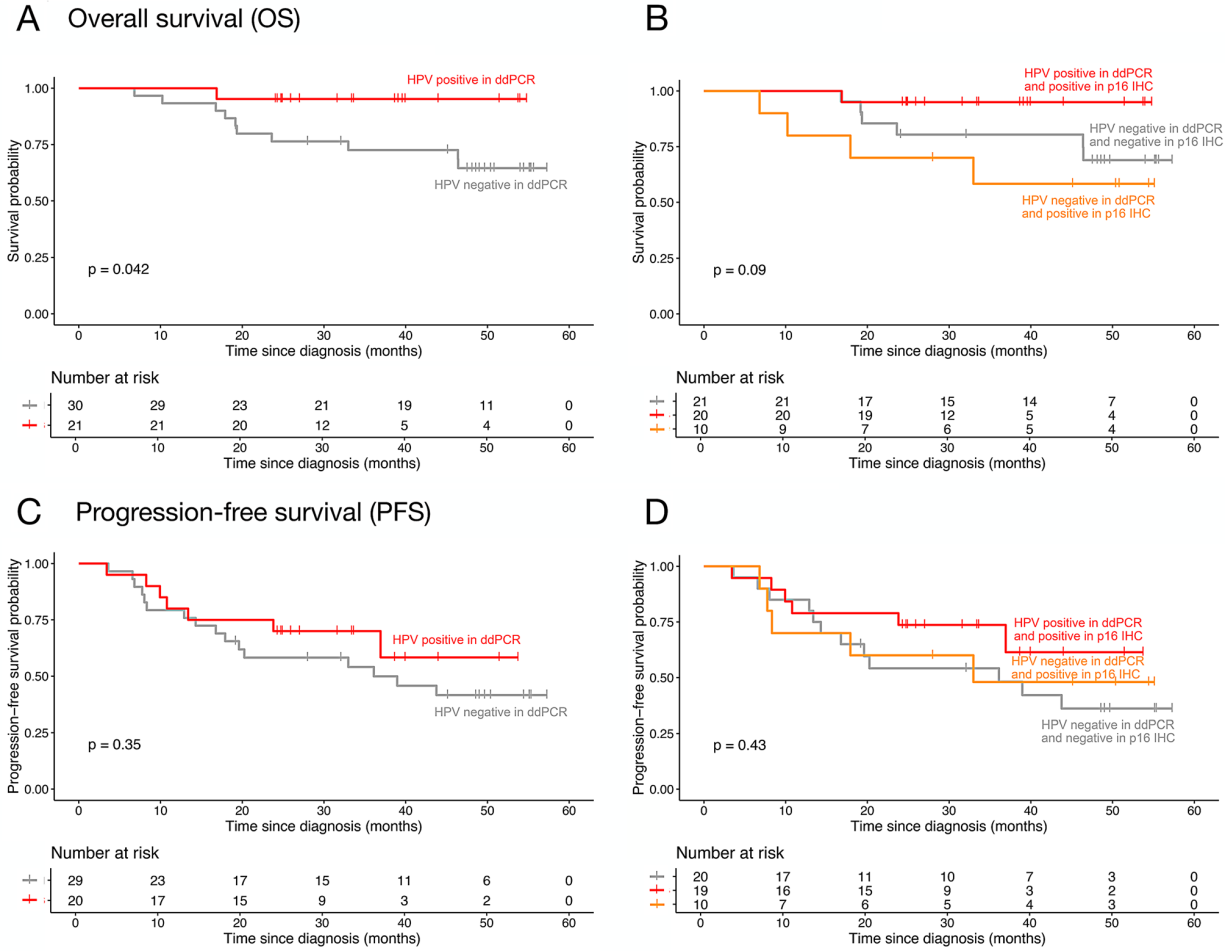
Kaplan-Meier survival analyses comparing HPV16 status by ddPCR and p16^INK4a^ immunohistochemistry. (**A**) overall survival (OS) stratified by ddPCR results. (**B**) OS stratified by concordant versus discordant results between ddPCR and IHC. (**C**) progression-free survival (PFS) stratified byddPCR results. (**D**) PFS stratified by concordance or discordance between ddPCR and IHC. Risk tables are shown below each plot; survival differences were evaluated using the log-rank test

We first analyzed OS in 51 patients with available outcome data, stratified by ddPCR-based HPV status in tumor gDNA (Fig. 3A). Patients classified as HPV16-positive (*n* = 21) showed significantly longer OS compared with HPV-negative cases (*n* = 30; *p* = 0.04, log-rank). Cox regression suggested improved survival in HPV16-positive patients (HR 0.16, 95% CI 0.02–1.22; *p* = 0.08), corresponding to an ~85% reduced risk of death. Next, we compared OS classification by ddPCR and p16^INK4a^ IHC (Fig. 3B). Patients positive by both methods had the best outcomes, while “questionable cases” (p16^INK4a^ IHC-positive but ddPCR-negative) showed worse survival than both concordant HPV-positive and HPV-negative groups, consistent with these being false-positive IHC results. PFS was evaluated in 49 patients with known progression status. ddPCR-based stratification showed improved PFS in HPV16-positive cases (Fig. 3C; *p* = 0.35, log-rank; HR 0.16, 95% CI 0.02–1.22; *p* = 0.08). Incorporating both ddPCR and p16^INK4a^ IHC (Fig. 3D), “questionable cases” again mirrored HPV-negative outcomes, further supporting the limited specificity of IHC alone. For comparison, Kaplan–Meier analyses of OS and PFS based solely on p16^INK4a^ IHC results in tumor tissue are provided in Supplementary Figure S2.

### Longitudinal ddPCR monitoring of HPV16 in liquid biopsies

**Fig. 4:**
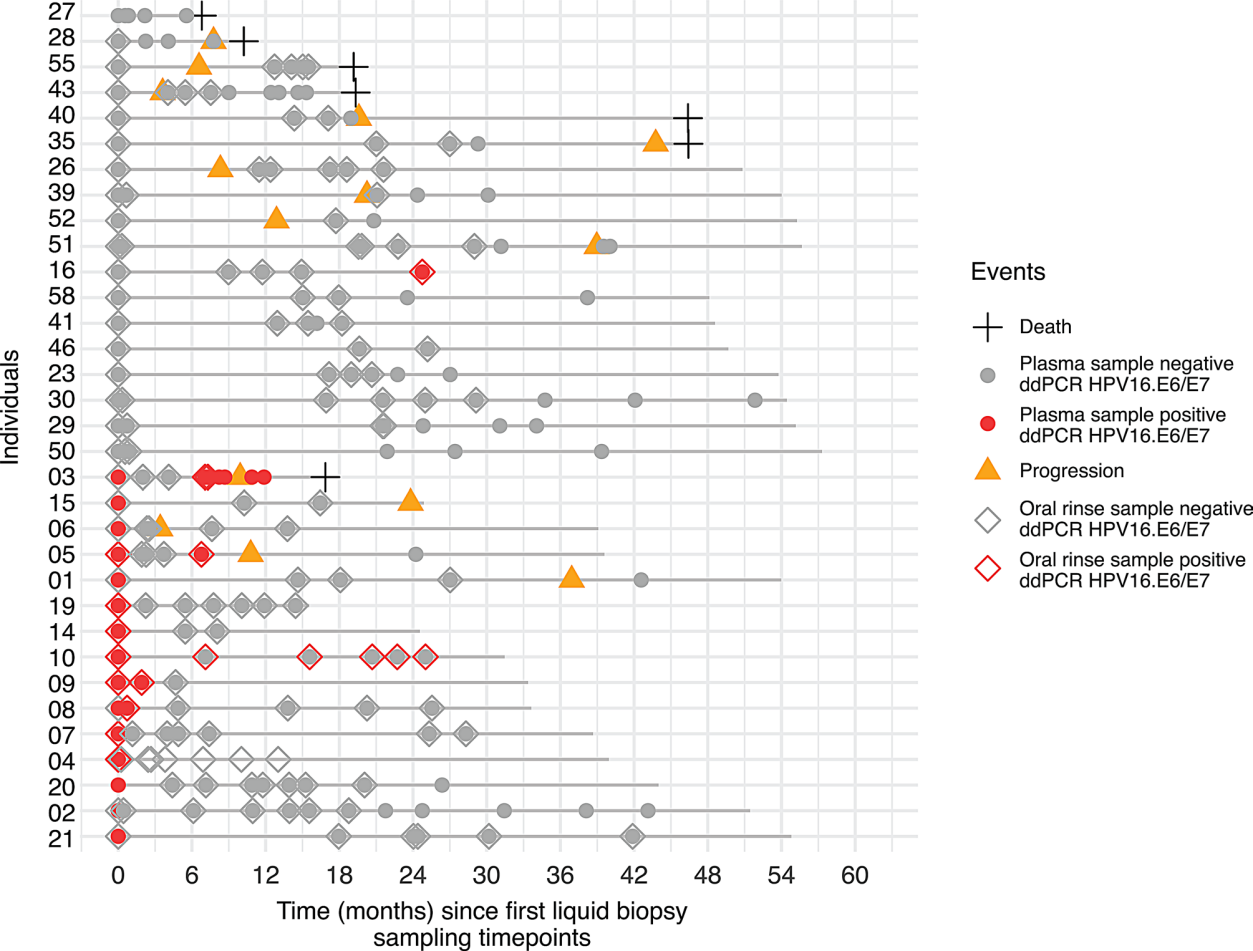
Longitudinal HPV16 monitoring using droplet digital PCR in liquid biopsies. Swimmer plots showing baseline and serial plasma and oral rinse results in 33 patients with follow-up samples. Initially HPV-positive patients cleared HPV DNA after surgery, with two showing re-detection of HPV DNA in plasma and oral rinse before clinical recurrence (lead times 87 and 122 days). Clinical events (progression, death) are annotated

To assess the prognostic value of ddPCR-based liquid biopsies, we analyzed longitudinal blood and oral rinse samples from 33 patients in the baseline & follow-up cohort. Each patient had a baseline liquid biopsy collected at or shortly before surgery and at least two postoperative samples. Swimmer plots were generated to visualize sampling time points and clinical outcomes, including progression and death events (Fig. 4). At baseline, 18 patients (55%) were HPV-negative; 10 of these (56%) developed progression or died during follow-up. The remaining 15 patients (45%) were HPV-positive in their baseline liquid biopsy. All subsequently converted to HPV-negative after surgery, indicating successful removal of the HPV-associated tumor (“HPV clearance”). Despite clearance, 5 patients (33%) later experienced progression. In two of them (ID03, ID05), HPV DNA reappeared in both plasma and oral rinse before recurrence (“loss of HPV clearance”), with lead times of 87 and 122 days. For the other three patients, no HPV re-detection occurred before progression, illustrating limitations of the approach.

## Discussion

Our ddPCR-based approach provides a robust and specific method for determining HPV status in primary tumor tissue from HNSCC patients. Using ddPCR to detect HPV16 E6/E7 in tumor-derived gDNA, 24/58 patients (41%) were HPV16-positive, supported by detection of E2 and HPV16 transcripts in cDNA. Ten p16^INK4a^-positive tumors lacked HPV16 DNA/RNA and likely represent false-positive IHC classifications. OPSCC-restricted analysis confirmed this pattern: among 14 p16-positive OPSCCs, only 9 showed HPV16 DNA, whereas 5 demonstrated p16 overexpression without molecular evidence of transcriptionally active HPV. Conversely, one p16-negative OPSCC was clearly HPV16-positive across tissue and liquid biopsy assays. These data indicate that ddPCR increases diagnostic specificity even within the oropharynx, where p16 IHC traditionally performs best. Outside the oropharynx, p16 overexpression may arise through HPV-independent mechanisms such as CDKN2A alterations, Rb pathway dysregulation, or senescence, underscoring the need for molecular confirmation. These observations are consistent with prior work showing reduced p16 specificity in non-oropharyngeal HNSCC [8] and p16 expression independent of transcriptionally active HPV [9]. The strong concordance of DNA- and RNA-based ddPCR findings, together with the poor survival of “questionable cases,” highlights ddPCR’s ability to identify biologically active HPV infection. A limitation is the HPV16-restricted assay panel; rare high-risk genotypes (e.g., HPV18/31/45) would require multi-genotype ddPCR or NGS-based HPV typing. DdPCR reliably detected HPV16 DNA in plasma and oral rinse collected at baseline, with detection rates closely matching tumor tissue results. All HPV-positive tissue cases were positive in at least one liquid biopsy, and all 44 healthy controls were negative, confirming analytical specificity. Plasma and oral rinse thus represent viable sample types for ddPCR-based HPV detection. Prior studies similarly showed that circulating HPV DNA in plasma correlates with tumor burden and treatment response in HPV-associated OPSCC [17], and that liquid biopsies are suitable for molecular profiling [13, 17]. Oral rinse may provide particular advantages due to mucosal HPV shedding, supported by spatial profiling data [18]. HPV RNA could not be detected in cDNA generated from liquid biopsy RNA, indicating that total RNA–based ddPCR from plasma or oral rinse is not feasible under current conditions and supporting circulating tumor DNA as the preferred analyte. These results support ddPCR-based HPV ctDNA as a specific prognostic adjunct in multimodal follow-up. Screening asymptomatic at-risk populations remains exploratory and requires validation in larger cohorts. Despite these promising findings, several limitations should be considered. First, the implementation of ddPCR in routine clinical diagnostics may currently be constrained by infrastructure requirements, reimbursement policies, and the need for specialized technical expertise, although increasing automation and broader adoption of digital PCR technologies are expected to improve scalability and cost-efficiency. Second, the lack of standardized protocols for oral rinse collection represents a potential source of variability, as differences in rinse volume and rinse duration may influence nucleic acid yield and assay sensitivity, highlighting the need for harmonized pre-analytical workflows. Finally, although liquid biopsy-based detection of HPV DNA may enable earlier identification of molecular relapse, the clinical benefit of such early detection remains to be assessed. It is currently unclear whether earlier intervention based on molecular findings translates into improved overall survival, and prospective interventional clinical trials are required to address this question.

## Conclusions

Altogether, our findings demonstrate that ddPCR offers a robust, sensitive, and highly specific platform for the detection and monitoring of HPV status in HNSCC patients. In primary tumor tissue, ddPCR outperforms p16^INK4a^ IHC in specificity while maintaining comparable sensitivity when applied to tumor-derived gDNA. Its ability to detect both the presence and transcriptional activity of the viral oncogenes HPV16 E6/E7 further enhances diagnostic precision and prognostic stratification. Our data also highlight the utility of liquid biopsy specimens for HPV detection. At baseline, plasma and oral rinse samples showed strong concordance with tumor-derived results and demonstrated no false-positive findings in healthy controls, supporting liquid biopsy as a viable alternative when tumor tissue is inaccessible or insufficient. Longitudinal analyses further indicate that serial ddPCR testing can provide clinically relevant information for monitoring treatment response. The observed patterns of HPV clearance following surgery and the loss of HPV clearance prior to radiologically confirmed recurrence underscore the potential of ddPCR-based assays as a prognostic tool in patient surveillance. OPSCC-specific analyses reinforce these conclusions. Even within the oropharynx where p16 IHC traditionally achieves its highest diagnostic performance ddPCR identified transcriptionally active HPV16 in only 9 of 14 p16-positive tumors, whereas 5 OPSCCs showed p16 overexpression without molecular evidence of HPV16. Conversely, ddPCR detected one p16-negative but HPV16-positive OPSCC case. These findings demonstrate that ddPCR improves diagnostic specificity and prevents misclassification even in the anatomical subsite where surrogate marker performance is strongest. High concordance across tumor tissue, plasma, and oral rinse in truly HPV16-driven OPSCC further supports ddPCR as a biologically grounded diagnostic and monitoring tool. However, given the small number of recurrence events with HPV re-detection in our cohort, further studies in larger, prospectively collected populations are required to refine the sensitivity of this approach and to elucidate biological determinants of variable HPV shedding. While ddPCR-based HPV detection in liquid biopsies is a promising adjunct for postoperative monitoring, its application for screening asymptomatic or unselected at-risk populations remains exploratory and requires rigorous evaluation before clinical implementation.

## Electronic supplementary material

Below is the link to the electronic supplementary material.

Supplementary Material 1

Supplementary Material 2

Supplementary Material 3

Supplementary Material 4

Supplementary Material 5

Supplementary Material 6

Supplementary Material 7

Supplementary Material 8

## Data Availability

Raw data from ddPCR experiments, including amplitude and cluster data, are available in the ZENODO repository (10.5281/zenodo.15731097). For data analysis and plotting, R scripts used in this manuscript were optimized and refined with the assistance of ChatGPT 4.0 (OpenAI, San Francisco, CA, USA).
