# Supplementary material for: Blood plasma and oral rinse liquid profiling for human papillomavirus in head and neck cancer – Unmasking false-positive p16 tissue cases and tracking disease dynamics
Source: J Transl Med. 2026 May 19;24:694. doi: 10.1186/s12967-026-08248-1 (PMC13192202; doi:10.1186/s12967-026-08248-1)
Supplement: Supplementary file 1 — Supplementary Material 1 [file 12967_2026_8248_MOESM1_ESM.pdf]

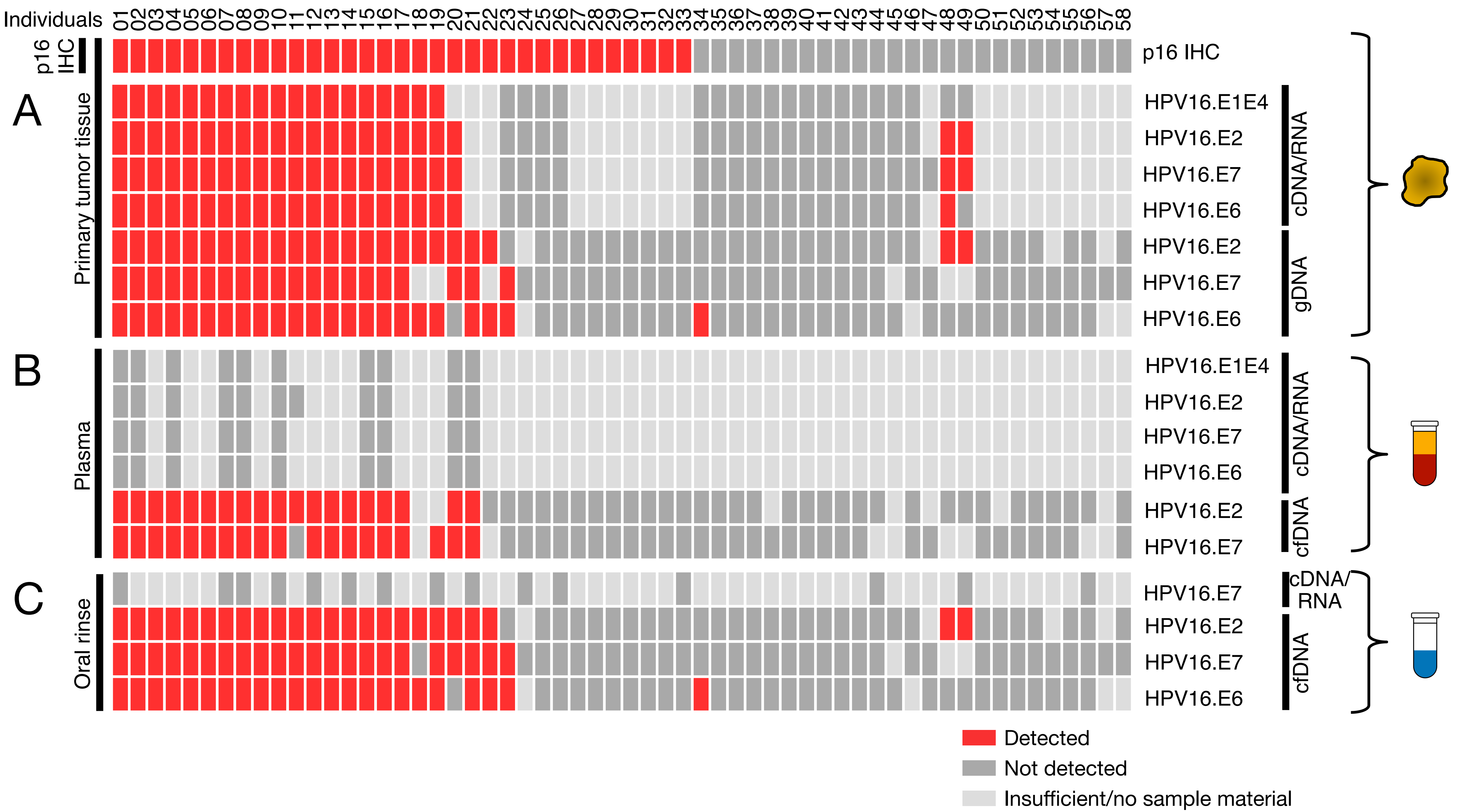

Supplementary Figure S1

The heatmap illustrates the results of droplet digital PCR (ddPCR) targeting HPV16 RNA transcripts (E6, E7, and E2) in total RNA isolated from liquid biopsy samples—specifically plasma (Panel B) and oral rinse (Panel C)—collected at baseline from HNSCC patients. For reference, Panel A includes transcript detection from primary tumor tissue cDNA, as shown in Figure 2. While HPV16 transcripts were readily detectable in tumor-derived cDNA, no HPV16 RNA was detected in any of the liquid biopsy samples. This indicates that total RNA extracted from plasma and oral rinse is not a feasible source for transcript-level HPV detection via ddPCR, likely due to low RNA abundance and degradation in biofluids. These findings highlight a fundamental limitation of RNA-based liquid biopsy approaches and support the exclusive use of DNA-based detection for non-invasive HPV testing.
