# Supplementary material for: Blood plasma and oral rinse liquid profiling for human papillomavirus in head and neck cancer – Unmasking false-positive p16 tissue cases and tracking disease dynamics
Source: J Transl Med. 2026 May 19;24:694. doi: 10.1186/s12967-026-08248-1 (PMC13192202; doi:10.1186/s12967-026-08248-1)
Supplement: Supplementary file 2 — Supplementary Material 2 [file 12967_2026_8248_MOESM2_ESM.pdf]

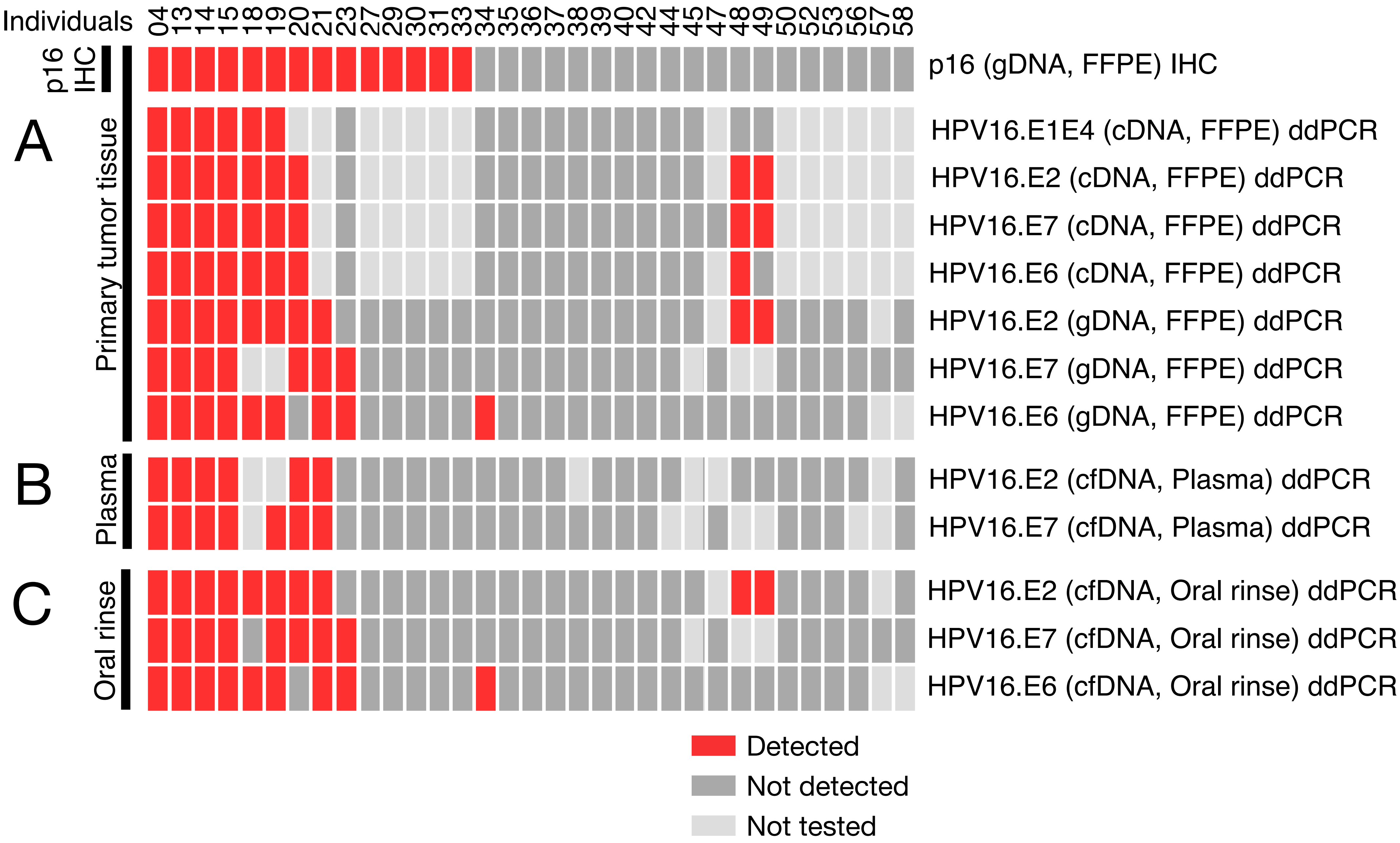

Supplementary Figure S2

OPSCC-restricted molecular and immunohistochemical detection of HPV16 across tumor tissue, plasma, and oral-rinse samples. This heatmap summarizes HPV16 detection exclusively in patients with oropharyngeal squamous cell carcinoma (OPSCC; n = 33). For each case, droplet digital PCR (ddPCR) results for HPV16 E6, E7, and E2 in genomic DNA (gDNA) extracted from FFPE tumor tissue are shown, alongside ddPCR detection of viral transcripts (E1^E4, E2, E6, E7) in cDNA derived from tumor RNA, indicating transcriptionally active infection. Detection of HPV16 E2 and E7 in circulating cell-free DNA (cfDNA) obtained from baseline plasma samples and of HPV16 E6, E7, and E2 in cfDNA from baseline oral-rinse samples is displayed in parallel. p16 immunohistochemistry (IHC) status is shown at the top for comparison. Red indicates detection, dark grey indicates absence, and light grey indicates assays not performed. The OPSCC-restricted visualization demonstrates high concordance between ddPCR-based HPV16 detection in tumor tissue and matched liquid biopsies, and highlights improved agreement between ddPCR and p16 IHC within this anatomically relevant subgroup.
