# Supplementary material for: Blood plasma and oral rinse liquid profiling for human papillomavirus in head and neck cancer – Unmasking false-positive p16 tissue cases and tracking disease dynamics
Source: J Transl Med. 2026 May 19;24:694. doi: 10.1186/s12967-026-08248-1 (PMC13192202; doi:10.1186/s12967-026-08248-1)
Supplement: Supplementary file 3 — Supplementary Material 3 [file 12967_2026_8248_MOESM3_ESM.pdf]

# HPV-KOHCIN – Sample Collection Tubes

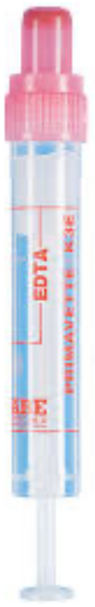


2x 4mL EDTA blood tube for the isolation of EDTA-Plasma (cfDNA) and buffy coat (gDNA)

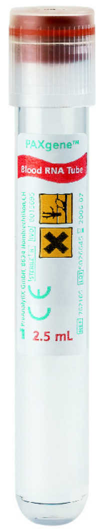


1x 2mL PAXgene blood tube for the isolation of total RNA from blood

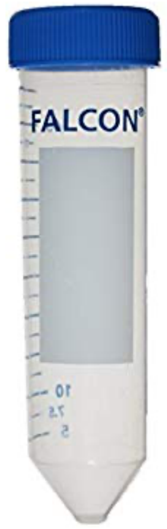


1x 50ml Falcon for oral rinse, 10mL 0.9% NaCl rinsing solution. (Isolation of saliva for cfDNA and total oral rinse RNA))

**Reagents and Kits Used:**

• • EDTA tubes

• • Sterile disposable pipettes

Collection of Plasma and Buffy Coat from EDTA Blood

1. Centrifugation:

Centrifuge the EDTA tubes at 2000xg for 15 minutes at 15°C.

2. Transfer Plasma:

Transfer all plasma into a new 15 mL tube without touching the buffy coat, using a sterile disposable pipette. Use a single-channel pipette (100–1000 µL) for the remaining volume.

3. Second Centrifugation:

Centrifuge the plasma again at 2000xg for 15 minutes at 15°C.

4. Record Plasma Volume:

Document the amount of plasma collected.

5. Aliquot Plasma:

Aliquot the plasma into 2 mL Eppendorf tubes. Document the number of aliquots.

6. Collect Buffy Coat:

Carefully collect the remaining blood cells (buffy coat) and transfer them into a 15 mL tube for later DNA extraction.

Refer to the protocol for Buffy Coat Collection.

**Reagents and Kits Used:**

• • 0.9% NaCl solution (sterile)

**Collection of Saliva and Cells from Saliva**

The patient should vigorously rinse and gargle with 10 mL of sterile 0.9% NaCl for 30–60 seconds. The entire volume is then expectorated into a sterile collection tube (e.g., 50 mL conical tube).

1. Centrifuge the collection tubes at 2000g for 15 minutes at 15°C.

2. Transfer the saliva supernatant into a fresh, labeled 15 mL tube.

Store the samples on ice or at -80°C for long term.

**Reagents and Kits Used:**

• • G-DEX erythrocyte lysis buffer (iNtRON Biotechnology)

• • Phosphate-buffered saline (PBS)

**Collection of Buffy Coat from Blood**

1. **Collect Buffy Coat:**

Carefully collect the remaining blood cells (buffy coat) using a 1 mL pipette and transfer them into a 15 mL Falcon tube.

2. **Add Lysis Buffer:**

Add 4 mL of G-DEX erythrocyte lysis buffer (iNtRON Biotechnology) (stored at 4°C) and resuspend thoroughly to ensure the erythrocytes and lysis buffer are well mixed.

3. **Incubate:**

Incubate for 10 minutes at room temperature (RT).

4. **Stop Reaction:**

Stop the reaction by filling the Falcon tube with PBS and mixing by inversion.

5. **Centrifuge:**

Centrifuge at 4000g for 10 minutes at 4°C.

6. **Remove Supernatant:**

Discard the supernatant and add another 4 mL of G-DEX erythrocyte lysis buffer.

7. **Second Incubation:**

Incubate for 10 minutes at RT.

8. **Stop Reaction Again:**

Stop the reaction by filling the Falcon tube with PBS and mixing by inversion.

9. **Second Centrifugation:**

Centrifuge again at 4000g for 10 minutes at 4°C.

10. **Check the Pellet:**

Remove the supernatant. A clear white cell pellet should be visible. If not, repeat the lysis step a third time.

11. **Resuspend Pellet:**

Resuspend the cell pellet in 1 mL of PBS and transfer it into a 2 mL Eppendorf tube.

12. **Final Centrifugation:**

Centrifuge at 4000g for 10 minutes at 4°C.

13. **Remove Supernatant:**

Carefully discard the supernatant.

14. **Next Steps:**

- If not proceeding immediately, place the pellet on dry ice.
- For subsequent **gDNA extraction**: Resuspend the pellet in 1 mL of DNAzol.

15. **Long term Storage:**

- Store pellets in DNAzol at 4°C.

**Reagents and Kits Used:**

• • Zymo Quick-DNA Miniprep Plus Kit

• • Qubit dsDNA HS Assay Kit

• • TapeStation HS DNA Tape

**Extraction of cfDNA from oral rinse Using Zymo Quick-DNA Miniprep Plus Kit**

1. **Time to Processing:**

Record the time elapsed between sample collection and processing.

2. **Thaw oral rinse:**

Thaw oral rinse samples on ice.

3. **Centrifugation:**

Centrifuge at 2000g for 15 minutes at 15°C.

4. **cfDNA Isolation Using Zymo Quick-DNA Miniprep Plus Kit:**

Follow the kit protocol (Version 1.2):

- **4.1**: Transfer **0.8 mL of the sample** into a 15 mL centrifuge tube.
- **4.2**: Add **0.8 mL of Biological Fluids & Cells buffer** (1:1 ratio to saliva).
- **4.3**: Add **80 µL of Proteinase K** (1:10 ratio to Biological Fluids & Cells).

Example: For 800 µL saliva, add 800 µL Biological Fluids & Cells + 80 µL Proteinase K.

- **4.4**: Vortex thoroughly.
- **4.5**: Incubate at 55°C for 10 minutes in a thermoshaker at 1000 rpm.
- **4.6**: Briefly centrifuge the mixture.
- **4.7**: Add an equal volume of Genomic Binding Buffer to the sample mixture (1:1 ratio).

Example: For 1680 µL sample mixture, add 1680 µL Genomic Binding Buffer.

- Vortex thoroughly.

5. **DNA Binding and Washing:**

- **5.1**: Apply **840 µL** of the sample mixture to the column and centrifuge at 12000g for 1 minute. Discard the flow-through. Repeat this step until the entire mixture has passed through the column.

- Transfer the column to a new tube.
- **5.2**: Add **400 µL DNA Pre-Wash Buffer** to the column and centrifuge at 12000g for 1 minute. Discard the flow-through.
- **5.3**: Add **700 µL g-DNA Wash Buffer** to the column and centrifuge at 12000g for 1 minute. Discard the flow-through.
- **5.4**: Add **200 µL g-DNA Wash Buffer** to the column and centrifuge at 12000g for 1 minute. Discard the flow-through.

6. **Elution:**

- Transfer the column to a labeled Eppendorf tube.
- Add **55 µL of Elution Buffer** to the column.
- Incubate at room temperature for 5 minutes.
- Centrifuge at 12000g for 1 minute.
- Repeat the elution step with the same eluate.

7. **Quality Control Analysis (QC):**

Perform QC using the following methods:

- **Qubit:** Use the Qubit® dsDNA HS Assay Kit.
- **TapeStation:** Use the HS DNA Tape.

8. **Long term storage:**

Store extracted cfDNA at -20°C

**Reagents and Kits Used:**

- • QIAamp Circulating Nucleic Acid Kit
- • Qubit dsDNA HS Assay Kit
- • TapeStation High Sensitivity D1000 ScreenTape

**Isolation of cfDNA from Plasma Using QIAamp Circulating Nucleic Acid Kit**

1. **Thaw Plasma:**

Thaw plasma on ice for approximately 1 hour.

2. **Centrifuge Plasma:**

After thawing, centrifuge at **2000g for 15 minutes at 15°C**.

3. **cfDNA Isolation:**

Use the Qiagen Circulating Nucleic Acid Isolation Kit to isolate cfDNA, adding **100 ng of carrier RNA (cRNA)** per sample. Use **2 mL of plasma per sample.**

4. **Kit Protocol:**

Follow the protocol provided with the kit. Ensure proper preparation of buffers for first-time use.

**Steps for cfDNA Isolation:**

5. **Prepare Samples:**

- Place **200 µL Proteinase K** into a 50 mL centrifuge tube.
- Add **2 mL plasma** to the tube.
- Add **1.6 mL ACL Buffer** (prepared with **100 ng cRNA per mL plasma**) to the plasma-Proteinase K mixture.
- Vortex for 30 seconds.

6. **Incubation:**

Incubate the mixture in a water bath at **60°C for 30 minutes.**

7. **Binding Preparation:**

Add **3.6 mL ACB Buffer** to the lysate and vortex for 15–30 seconds.

Incubate on ice for 5 minutes.

8. **Vacuum Setup:**

- Turn on the vacuum pump.
- Attach the VacConnector to the QIAvac 24 Plus system.
- Attach columns to the connector and place a **20 mL tube extender** onto each column.

9. **Load Sample:**

- Transfer the sample into the **20 mL tube extender.**
- Allow the sample to pass completely through the column using the vacuum pump (approximately 3–5 minutes).

10. **Remove Extender:**

- Once the sample has passed through, turn off the vacuum pump and release the pressure.
- Remove the tube extender from the column.

11. **Wash Steps:**

- Add **600 µL ACW1 Buffer** to the column. Do not close the column cap. Turn on the vacuum pump.
- Allow the buffer to pass completely, then turn off the pump and release pressure.
- Add **750 µL ACW2 Buffer** to the column. Repeat the process.
- Add **750 µL of 100% ethanol** to the column and allow it to pass completely.

12. **Dry Column:**

- Close the column caps and transfer to a **collection tube.**
- Centrifuge at **20,000g for 3 minutes.**

13. **Elution:**

- Place the column in a fresh collection tube. Incubate at **56°C for 10 minutes** in a heating block.
- Transfer the column to a labeled 1.5 mL Eppendorf tube.

14. **Elution Volume:**

- Add **72 µL of Elution Buffer** in two steps: **1×40 µL** and **1×32 µL.**
- Incubate at room temperature for 3 minutes.
- Centrifuge at **20,000g for 1 minute.**

15. **QC Analysis:**

- Perform quality control using the **Qubit® dsDNA HS Assay Kit** and **TapeStation High Sensitivity D1000 ScreenTape.**

**Reagents and Kits Used:**

• • DNAzol Reagent

• • 100% and 70% Ethanol

• • Qubit DNA BR/HS Assay Kit

**Extraction of gDNA from Buffy Coat**

1. **Collect Buffy Coat:**

Carefully collect the remaining blood cells (Buffy Coat) and store them in 2 mL Eppendorf tubes at -80°C for later DNA extraction or proceed directly with the process.

2. **Time to Processing:**

Record the time elapsed between Buffy Coat collection and processing:

3. **Preparation of Buffy Coat:**

- If the Buffy Coat is already stored in 1 mL of DNAzol at 4°C, remove it from the refrigerator and allow it to reach room temperature (RT) (approximately 5–15 minutes).
- If the Buffy Coat is stored at -80°C, thaw the samples on ice, then resuspend them in 1 mL of DNAzol, ensuring thorough mixing.

4. **Incubation:**

Incubate the samples for 10 minutes at RT.

5. **Add Ethanol:**

Add 500 µL of 100% ethanol and vortex thoroughly until no visible phases remain.

6. **Second Incubation:**

Incubate for 5 minutes at RT.

7. **Centrifugation:**

Centrifuge the samples at 2500g for 2 minutes at RT.

8. **Remove Supernatant:**

Carefully discard the supernatant.

9. **Wash Pellet:**

Wash the pellet with 500 µL of 70% ethanol. Gently swirl the pellet but do not fully resuspend it.

10. **Second Centrifugation:**

Centrifuge again at 2500g for 2 minutes at RT.

11. **Repeat Wash Step:**

Remove the supernatant and repeat the washing step with 70% ethanol.

12. **Final Supernatant Removal:**

Discard the supernatant completely.

13. **Dry Pellet:**

Allow the pellet to air dry, or dry it in a heating block at approximately 37°C.

14. **Resuspend Pellet:**

Depending on the pellet size, dissolve it in 50–100 µL of RNase/DNase-free water.

15. **Heat to Dissolve:**

Incubate the sample in a heating block at 65°C for 1 hour to ensure complete dissolution.

16. **Measure DNA Concentration:**

Measure the DNA concentration using Qubit (DNA BR/HS assay). Record the results in the DNA_RNA concentration table within the ddPCR lab book or the designated Qubit-DNA_RNA concentration table.

17. **Storage:**

Store the extracted DNA at -20°C.

**Reagents and Kits Used:**

• • PAXgene Blood RNA Kit

• • Qubit RNA BR Assay Kit

• • TapeStation RNA ScreenTape

Extraction of cfRNA from Plasma using PAXgene Blood RNA Kit

**1** Centrifuge the PAXgene Blood RNA Tubes (BRT) for 10 minutes at 4000g in a swinging-bucket rotor.
Incubate blood samples in the BRTs for **at least 2 hours at room temperature (15–25°C)** prior to centrifugation to ensure complete lysis of blood cells!

**2** Remove the supernatant (by pipetting – start with a serological pipette, finish with a 1000µl pipette), being cautious as the pellet may easily detach.
Resuspend the pellet in 4 ml RNase-free water (RNFW), then close the tube with a new BD Hemogard safety cap (included in the kit).

**3** Resuspend pellet by briefly vortexing, then centrifuge at 4000g for 10 minutes.
Remove the supernatant (pipette carefully – the pellet may loosen easily).

**4** Add 350µl Resuspension Buffer (BR1), vortex well until the pellet is fully dissolved.

**5** Transfer the sample with a pipette to a 1.5 ml microcentrifuge tube (MCT).

**6** Add 300µl Binding Buffer (BR2) and 40µl Proteinase K (PK), vortex for 5 seconds.
**Do not pre-mix BR2 and PK – add them individually to the sample.**

**7** Incubate for 10 minutes at 55°C, shaking at 400–1400 rpm in a heat block.

**8** Transfer the lysate directly into a PAXgene Shredder spin column (PSC, purple), centrifuge for 3 minutes at 20,000g.

**9** Carefully transfer the entire supernatant — without disturbing the pellet — to a new 1.5 ml microcentrifuge tube (MCT).

**10** Add 350µl ethanol (96–100%, analytical grade), mix (vortex), and briefly centrifuge (no more than 1–2 seconds – longer spinning can reduce total RNA yield).

**11** Transfer 700µl of the sample into a PAXgene RNA spin column (PRC, red), centrifuge for 1 minute at 8000g.
Place the column into a 2-ml processing tube (PT), discard the old one.
Transfer the remaining sample to the same spin column, centrifuge 1 minute at 8000g, then place the column in a new PT and discard the previous one.

**12** Add 350µl Wash Buffer 1 (BR3) to the spin column, centrifuge for 1 minute at 8000g, transfer the column to a new PT and discard the old one.

**13** For each sample, prepare a DNase I incubation mix by combining 10µl DNase I stock solution (RNFD) with 70µl DNA digestion buffer (RDD) in a 1.5 ml MCT.

Gently flick to mix and briefly centrifuge. **Do not vortex!**

*Example for 10 samples: 100µl RNFD + 700µl RDD*

**14** Pipette 80µl of the DNase I mix directly onto the membrane in the spin column. Incubate for 15 minutes at room temperature (20–30°C).

**15** Add 350µl Wash Buffer 1 (BR3) to the spin column, centrifuge for 1 minute at 8000g, transfer to a new PT and discard the old one.

**16** Add 500µl Wash Buffer 2 (BR4), centrifuge for 1 minute at 8000g, transfer to a new PT and discard the old one.

**17** Add another 500µl of BR4, centrifuge for 3 minutes at 8000g, transfer to a new PT and discard the old one.

**18** Centrifuge for 1 minute at 8000g, then place the spin column in a fresh 1.5 ml Eppendorf tube. Discard the processing tube.

**19** Pipette 40µl Elution Buffer (BR5) directly onto the membrane of the spin column, centrifuge for 1 minute at 8000g to elute the RNA.

**20** Incubate the eluate at 65°C in a heat block for 5 minutes **without shaking**, then immediately place on ice.

**5. QC Analysis (Qubit/TapeStation):**

**Qubit:** Use Qubit® RNA BR Assay Kit

**TapeStation:** Use RNA ScreenTape, RNA ScreenTape Sample Buffer, and Ladder
(Protocols are available in the lab)

**Reagents and Kits Used:**

• • Qiagen miRNeasy Mini Kit (#217004)

• • Qubit RNA BR/HS Assay

• • PerfeCTa DNase I, Quantabio (#95150-100)

• • qScript XLT cDNA SuperMix

1. RNA Isolation from oral rinse using Qiagen miRNeasy Mini Kit
2. Isolation is performed using the miRNeasy Mini Kit (#217004)
3. add 700 µl QIAzol Lysis Reagent, mix thoroughly (use pipette and vortex)
4. Incubate for 5 minutes at room temperature (15–25°C)
5. Add 140 µl chloroform, vortex well (until no phases are visible anymore) and incubate for 3 minutes at RT
6. Centrifuge at 12,000g for 15 minutes at 4°C
7. Transfer the upper aqueous phase to a new microcentrifuge tube (Eppi)
8. Add 1.5 volumes (525 µl) of 96–100% ethanol
9. Transfer 700 µl of the sample to the spin column, centrifuge at 8000g for 15 seconds
10. Repeat step 10 with the remaining sample
11. Add 700 µl RWT buffer, centrifuge at 8000g for 15 seconds, discard flow-through
12. Add 500 µl RPE buffer, centrifuge at 8000g for 15 seconds, discard flow-through
13. Add 500 µl RPE buffer, centrifuge at 8000g for 2 minutes, discard flow-through
14. Place the column in a new collection tube and centrifuge at 20,000g for 1 minute
15. Transfer column to a labeled Eppi and elute RNA in 50 µl RNase-free water, centrifuge at 8000g for 1 minute
16. Measure RNA concentration using Qubit (RNA BR/HS Assay)
17. Either perform DNase digestion (using PerfeCTa DNase I, Quantabio #95150-100) or store at -80°C
18. Perform cDNA synthesis using qScript XLT cDNA SuperMix
    - Reaction volume: 20 µl
    - Use 1 µg RNA per cDNA synthesis reaction

**Reagents and Kits Used:**

• • PerfeCTa DNase I, Quantabio (95150-100)

• • qScript XLT cDNA SuperMix, Quantabio (733-2374)

• • TapeStation High Sensitivity D1000

• • Qubit HS DNA Assay

1. DNase Digestion and cDNA Synthesis with cfRNA or RNA

2. Perform DNase Digestion by setting up reactions for each sample according tot he tables below. Using DNase digestion with PerfeCTa DNase I, Quantabio (95150-100):

3. Components
Volume for 35 µl reaction:

4. 10x Stop Buffer: 3.5 µl

5. Final Volume: 38.5 µl

6. Cycler Program

7. Perform cDNA Synthesis with qScript XLT cDNA SuperMix, Quantabio (733-2374, VWR) by setting up reactions as shown below:

8. Final Volume: 23.125 µl

9. cDNA synthesis with qScript XLT cDNA SuperMix, Quantabio (733-2374, VWR)

10. 3. Perform Quality Control using TapeStation: High Sensitivity D1000 ScreenTape; High Sensitivity D1000 Reagents and Ladder and Qubit: Qubit HS DNA Assay

**Reagents and Kits Used:**

• • BioRad ddPCR SuperMix (no dUTP, #1863023)

• • HaeIII Enzyme (NEB, #R0108L)

• • ddPCR Consumables (plates, oil, cartridges, etc.)

• • Qubit and droplet reader systems

**Setting up ddPCRs**

**1. Required Materials**

- BioRad SuperMix (-20°C or thawed at 4°C; no dUTP; #1863023)
- PrimerMix (18 µM)
- Probe FAM/HEX (5 µM)
- Digestion enzyme HaeIII (stored at -20°C/-80°C depending on assay, NEB; # R0108L)
- $H_2O$ (DNase/RNase-free)
- TE buffer (for diluting PrimerMix/probes/gBlock)
- gBlock (positive/negative control)
- gDNA (negative controls)
- C1000 Touch Thermal Cycler with 96-Deep Well Reaction Module; #1851197
- BioRad Automated Droplet Generator; #1864101
- BioRad QX Droplet Reader; #1864003
- PX1 PCR Plate Sealer; #1814000
- Pierceable Foil Heat Seal, 100/PK; #1814040
- Pipet Tips for AutoDG, 20/PK; #1864120
- ddPCR Reader Oil, 2x1L; #1863004
- ddPCR Plates 96-Well, Semi-Skirted, 25/BX; #12001925
- DG32 AutoDG Cartridges, 30/PK; #1864108
- Waste Bins for AutoDG, 10/PK; #1864125
- AutoDG Oil for Probes, 1x140ml; #1864110
- ddPCR Buffer Control for Probes; #1863052

- Ice block for plate (freezer #1, compartment 1)

| Sample Input ddPCR | | |
|---|---|---|
| | Input | H2O |
| H2O | 6.3µl | |
| gBlock | $1x10^3$ copies dilution in 6.3 µl | |
| gDNA | 6,3µl | |
| cDNA | 6,3µl | |
| cfDNA | 6,3µl | |

**2. Pipetting ddPCR**

1. Thaw all required reagents.
2. The enzyme must be diluted to 5U with the appropriate diluent buffer.
3. Prepare the master mix according to the table, vortex thoroughly.

| Master Mix per reaction well<br><br>**HPV16/EIF2C1** | **1,05** |
|---|---|
| **2x ddPCR supermix for probes (no dUTP) (BioRad)** | 10,50 µl |
| **Diluted enzyme (HaeIII, 5U) (NEB)** | 1,05 µl |
| **20x target primer mix [18 µM] (900 nM)** | 1,05 µl |
| **20x target probe (FAM) [5 µM] (250 nM)** | 1,05 µl |
| **20x reference probe (HEX) [5 µM] (250 nM)** | 1,05 µl |
| Volume MM | **14,70 µl** |

4. Pipette 14.7 µl into each well (pmTERT assay BioRad requires 15 µl – an exception).

5. All eight wells in a column must be filled. If a well cannot be filled with master mix/probe, it must be filled with 21 µl of buffer (ddPCR Buffer Control for Probes).

6. Before pipetting samples into the wells, verify that each well contains the master mix or if master mix has been pipetted twice in one well.

7. Follow the input table (page 2) for input volume and concentration; confirm details with Silvia/Christof.

8. Pipette $H_2O$ (in all wells), gDNA/cDNA control, samples, gBlock, and, if necessary, buffer into wells.

9. Once ddPCR is pipetted, place foil on the plate, seal it with the plate sealer.

10. Briefly vortex and centrifuge the plate.

11. Place the plate into the droplet generator.

**3. Droplet Generation**

1. Fill the generator from back to front (cartridges, tips, empty plate on ice block, sample plate).

2. Use the droplet generator touch screen to select the required number of rows. Ensure everything is correctly placed in the generator.

3. Start droplet generation (approximately 50 minutes per plate).

4. During droplet generation, prepare the run protocol for the droplet reader (see section 5).

**4. ddPCR Run**

1. Remove generated droplets from the droplet generator, seal the plate again with foil.

2. Place the ddPCR plate into the thermal cycler, select the program (there are currently three different programs; see table).

3. Run the PCR according to the following protocol:

| | | |
|---|---|---|
| | 95° | 10 min |
| | 94° | 30 s |
| 40x | 60°C | 30 s |
| | 60°C | 30 s |
| | 98°C | 10 min |
| | 4°C | ∞ |

4. After the PCR, store the ddPCR plate overnight at 4°C if desired (based on experience, more droplets are present after overnight storage).

**5. Droplet Reading**

1. Turn on the droplet reader (switch at the back right) and start the QuantaSoft program.
2. Load the ddPCR protocol into QuantaSoft and wait 10–15 minutes until the plate can be placed into the reader.
3. Insert the ddPCR plate into the droplet reader and select the assay.
4. Start the analysis (including image processing); one plate takes approximately 2 hours.
5. Once the plate is read, discard it and turn off the reader.
